# Macroalgal-Derived Bioactive Compounds as Anti-Inflammatory and Antioxidant Ingredients for Food and Nutraceutical Industry: Mechanisms, Functional Applications, and Challenges

**DOI:** 10.3390/md24070254

**Published:** 2026-07-22

**Authors:** Sandra Pedisić, Josipa Dukić, Ena Cegledi, Ana Dobrinčić, Zoran Zorić, Zdenka Pelaić, Ivona Elez Garofulić, Maja Repajić, Verica Dragović-Uzelac

**Affiliations:** 1 Centre for Food Technology and Biotechnology, University of Zagreb Faculty of Food Technology and Biotechnology, 23000 Zadar, Croatia; sandra.pedisic@pbf.unizg.hr (S.P.); zpelaic@pbf.hr (Z.P.); 2 University of Zagreb Faculty of Food Technology and Biotechnology, 10000 Zagreb, Croatia; ecegledi@pbf.hr (E.C.); ana.dobrincic@pbf.unizg.hr (A.D.); ivona.elez@pbf.unizg.hr (I.E.G.); maja.repajic@pbf.unizg.hr (M.R.); vdragov@pbf.hr (V.D.-U.); 3 Department of Ecology, Agronomy and Aquaculture, University of Zadar, 23000 Zadar, Croatia; zzoric@unizd.hr

**Keywords:** macroalgae, polyphenols, pigments, polysaccharides, antioxidant and anti-inflammatory activity, extraction technologies, encapsulation, functional applications in foods and nutraceuticals, regulatory aspects

## Abstract

Marine-derived bioactive compounds have attracted considerable attention as functional ingredients for food and nutraceutical applications due to their various biological activities. Among marine resources, macroalgae represent a sustainable and abundant source of structurally diverse bioactive compounds, including polyphenols, pigments, and polysaccharides. This review provides a comprehensive overview of macroalgal bioactive compounds, with particular emphasis on their sources, the environmental and seasonal factors influencing their composition, chemical classification and characteristics, extraction technologies, biological properties and food and nutraceutical applications. Particularly, attention is given to the molecular mechanisms underlying their antioxidant and anti-inflammatory effects, including radical scavenging, metal chelation, modulation of endogenous antioxidant defense systems, and regulation of key signaling pathways involved in inflammation. Green extraction techniques and encapsulation strategies for improving the stability, bioavailability, and functionality of macroalgal bioactives are critically discussed. Current applications in foods and nutraceutical products are reviewed alongside the major challenges related to biomass variability, large-scale production, standardization, and regulatory compliance. Overall, macroalgal bioactive compounds represent a promising class of sustainable health-promoting ingredients, and continued advances in cultivation, processing, extraction technologies, formulation, and regulatory frameworks will be essential to support their broader industrial utilization.

## 1. Introduction

### 1.1. Background

In recent decades, marine environments have emerged as a promising source of structurally unique and biologically potent natural products with significant potential for use in the food, pharmaceutical, cosmetic, and nutraceutical industries. Among marine resources, macroalgae or seaweeds have attracted particular attention due to their abundance, sustainability, and rich content of bioactive compounds (BACs). In addition, macroalgae play important ecological roles in primary production, nutrient cycling, and habitat formation in coastal environments [1]. Adaptation to highly variable environmental conditions, such as fluctuations in salinity, temperature, light availability, nutrient concentrations, and pressure gradients, has driven the evolution of specialized metabolic pathways that enable macroalgae to survive under environmental stress. Consequently, macroalgae produce various BACs, including polyphenols, pigments, polysaccharides, and other metabolites that contribute to ecological adaptation and stress tolerance.

### 1.2. Research Significance

Many of these compounds exhibit potent antioxidant and anti-inflammatory properties. Numerous studies have shown that they can modulate oxidative stress and inflammatory processes through multiple mechanisms, including scavenging free radicals and other reactive species, regulating endogenous antioxidant defense systems, and influencing key cellular signaling pathways associated with inflammation [2,3]. Moreover, the composition of pigments, cell wall structure, and polysaccharide storage vary according to the taxonomic group of macroalgae (brown, red, or green algae) [4,5]. As the biochemical composition of macroalgae is highly dynamic and influenced by environmental and seasonal factors [6], the quantity and quality of BACs may vary considerably among species and harvesting conditions. Understanding these factors is essential for optimizing biomass cultivation and harvesting strategies, and for ensuring reproducible bioactive profiles suitable for industrial applications. Once biomass has been obtained, efficient extraction and purification of BACs become critical steps in their valorization and industrial use. Conventional solvent-based extraction (CE) methods are increasingly being combined with or replaced by advanced, environmentally friendly extraction technologies, e.g., microwave-assisted extraction (MAE), ultrasound-assisted extraction (UAE), and supercritical fluid extraction (SFE), which offer advantages in extraction efficiency, selectivity, reduced solvent consumption, shorter processing times, and improved sustainability [7,8]. Efficient extraction strategies are closely related to the chemical diversity and structural complexity of macroalgal BACs. These bioactives range from low- to high-molecular-weight compounds and often have unique structural features, such as halogen substitutions, specific sulfation patterns, and complex stereochemistry, which are associated with distinct mechanisms of action and enhanced antioxidant and anti-inflammatory properties [3,9,10]. Their chemical characterization is crucial for understanding structure–activity relationships and health-promoting effects. Given the central role of oxidative stress and chronic inflammation in the development of many non-communicable diseases, the pleiotropic mechanisms of action of macroalgal BACs have attracted considerable interest for their use as multifunctional ingredients in preventive nutrition and therapeutic applications [11,12]. However, despite their promising bioactivity, the practical application of many macroalgal bioactives is often limited by chemical instability and low bioavailability due to poor solubility, susceptibility to oxidation, or degradation during processing and storage. Encapsulation technologies have therefore emerged as valuable tools for protecting sensitive compounds from environmental stress, improving stability and bioavailability, and facilitating the incorporation of marine BACs into complex food and nutraceutical matrices. Various encapsulation approaches, including ionic gelation, chitosan-based nanoparticles, nanoemulsions, liposomes, freeze-dried microcapsules, and electrosprayed microcapsules, are being investigated to maximize the functional performance of macroalgal bioactives [13,14,15]. The choice of encapsulation technique depends on the chemical nature of the BAC and the intended application.

### 1.3. Aim and Scope of Review

Nevertheless, several challenges remain, including variability in raw materials, production costs, large-scale processing, regulatory compliance, and the need for further validation of biological efficacy and safety [13]. In this context, the present review offers a comprehensive overview of macroalgal-derived BACs, including polyphenols, pigments, and polysaccharides, emphasizing their sources, chemical classification, extraction techniques, and mechanisms of antioxidant and anti-inflammatory activity. It also addresses encapsulation strategies to enhance stability and functionality, explores functional applications in the food and nutraceutical industries, and discusses current challenges, regulatory aspects, and future perspectives. This review integrates recent scientific advances and technological developments to provide a comprehensive, up-to-date assessment of macroalgal-derived health-promoting compounds, evaluate their opportunities and limitations for sustainable use, and identify key challenges and future research directions for industrial applications.

### 1.4. Literature Search Strategy

The literature search was conducted using the Web of Science, Scopus, Google Scholar, and PubMed databases by applying various combinations of relevant keywords and Boolean operators to maximize the retrieval of pertinent studies. The identified records were screened for relevance to the scope of this review, with priority given to original research articles reporting experimental data on macroalgal composition and bioactive compounds, their chemical classification, extraction technologies, antioxidant and anti-inflammatory activities, encapsulation strategies, functional applications, and regulatory aspects. This review primarily focuses on studies published between January 2016 and June 2026. However, earlier seminal publications were also included where necessary to provide historical context and foundational knowledge. The selection process involved an initial screening of titles and abstracts, followed by a full-text evaluation of potentially relevant articles to ensure the inclusion of scientifically rigorous, high-quality, and relevant studies.

## 2. Marine Macroalgae as Sources of Bioactive Compounds

Macroalgae are a diverse group of multicellular marine organisms classified into three major phyla based on pigmentation: *Phaeophyceae* (brown); *Chlorophyta* (green); and *Rhodophyta* (red), each exhibiting distinct bioactive profiles [16]. According to AlgaeBase [17] red algae are the most diverse macroalgal group, comprising approximately 6000–7000 species, followed by brown algae with 2000–2200 species and green macroalgae with 900–1500 species. These groups are predominantly marine and include several ecologically and economically important orders: *Gigartinales*, *Gracilariales*, and *Ceramiales* in red algae; *Fucales*, *Laminariales*, and *Dictyotales* in brown algae; and *Ulvales* and *Bryopsidales* in green algae. Across these phyla, macroalgae are abundant sources of diverse BACs, including polyphenols, pigments, polysaccharides, lipids, proteins, and minerals [18]. Their sessile adaptation to dynamic marine conditions promotes the synthesis of secondary metabolites with pronounced antioxidant and anti-inflammatory properties, making them valuable ingredients for food and nutraceutical applications [19]. In addition, the increasing global production of cultivated algae and the approval of several macroalgal species as novel foods in the EU highlight their growing commercial relevance [20]. Nevertheless, the composition and concentration of BACs vary considerably among species and environmental conditions, highlighting the need to understand these factors to ensure biomass quality and support industrial utilization.

### 2.1. Environmental Factors Influencing Biochemical Composition of Marine Macroalgae

**Figure 1:**
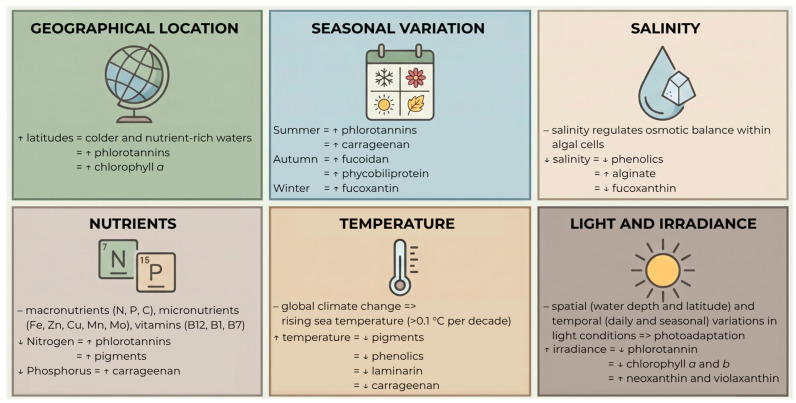
Environmental factors influencing the biochemical composition of marine macroalgae.

The bioactive composition of macroalgae shows substantial variability due to abiotic and biotic factors, species-specific traits, and post-harvest processing conditions (Figure 1). Abiotic factors such as geographical origin, seasonality, nutrient availability, temperature, light, UV radiation, and salinity strongly influence metabolic profiles, while biotic interactions, including grazing pressure and microbial associations, further modulate the synthesis of BACs [21]. In addition, inherent differences in metabolism and physiology among species also contribute to considerable variation in compound composition, even under similar environmental conditions. Furthermore, extraction conditions can significantly affect the yield and profile of detected compounds, as discussed in Section 4. Taken together, these environmental and biological factors drive the dynamic regulation of macroalgal metabolism. These natural fluctuations arise because macroalgae, as sessile organisms, activate sophisticated defense mechanisms in response to environmental stressors, producing secondary metabolites, such as polyphenols, pigments, and polysaccharides, when antioxidant protection or structural reinforcement is required [22]. This variability presents both challenges and opportunities for industrial applications, as understanding these patterns enables targeted harvest strategies, strain selection, and cultivation optimization to achieve consistent yields of nutraceutical-grade compounds.

#### 2.1.1. Geographical Location

The geographical location of macroalgae influences their biochemical profiles due to differences in environmental conditions such as water temperature, salinity, and nutrient regimes. Studies have shown that the same species collected from different regions can exhibit significant variation in phenolic, pigment and polysaccharide content. For example, brown algae from higher latitudes, characterized by colder and nutrient-rich waters, often contain higher levels of phlorotannin polyphenols compared to those from warmer regions [23]. In a study of nine Antarctic brown and red seaweed species along a latitudinal gradient from the South Shetland Islands (~62° S) to Yalour Island (~65° S), chlorophyll concentration tended to increase with latitude [24]. Furthermore, significant differences in the yield of the sulfated polysaccharide fucoidan have been observed in *Sargassum ilicifolium* collected from five regions within the same sea area of Taiwan [25].

#### 2.1.2. Seasonal Variation

Besides geographical location, seasonal variation is a major factor influencing macroalgal biochemical composition, reflecting the integrated effects of changes in temperature, irradiance, and nutrient availability throughout the year. In brown algae, phlorotannins typically reach their highest concentrations during summer. For example, in *Ascophyllum nodosum* levels increased from 0.6% in February to 2.2% in July [26], and in *Fucus spiralis* total phenolic content (TPC) increased from 49.170 mg GAE/g in spring to 308.634 mg GAE/g in summer [27]. Interestingly, a recent study showed that phlorotannin content in storm-cast *Ascophyllum nodosum* exceeded by 10% during July–August, displaying less pronounced seasonal fluctuations than in freshly harvested algae and highlighting the potential of both fresh and storm-cast biomass as sustainable sources of phlorotannins for food, cosmetic, and pharmaceutical application [28]. Similarly, in green algae such as *Ulva lacinulata* and *Codium tomentosum*, chlorophyll and carotenoid contents increase from winter to summer, reaching peak levels under enhanced light and nutrient availability, with chlorophyll content increasing by 33% in *U. lacinulata* and 57% in *C. tomentosum* [29]. In contrast, the carotenoid pigment fucoxanthin in the brown algae *Sargassum horneri* and *Cystoseira hakodatensis* reached its maximum concentration during winter [30]. Some red algae, such as *Palmaria palmata*, show increased phycobiliprotein accumulation (pigment–protein complexes) in autumn (September–November), while carotenoids and chlorophylls reach their maximum concentration in winter and minimum in summer, reflecting adaptation to reduced light conditions [31]. In four brown algae (*Alaria esculenta*, *Fucus distichus*, *Laminaria digitata*, and *Saccharina latissima*) and one red alga (*Palmaria palmata*), chlorophyll and the concentrations of the carotenoids fucoxanthin and violaxanthin were higher in August, whereas lutein and zeaxanthin levels were higher in June [32]. Seasonal effects are also evident in polysaccharide composition, as fucoidan content in brown algae *Fucus serratus*, *Fucus vesiculosus*, *Ascophyllum nodosum*, *Cystoseira barbata*, *Cystoseira compressa*, and *Sargassum vulgare* increases during autumn growth periods and declines in spring and winter [33,34] while the highest carrageenan content in red algae *Mastocarpus stellatus* was identified in August [35].

#### 2.1.3. Nutrient Availability

Macroalgal growth and metabolic activity depend on the availability of macronutrients (e.g., nitrogen, phosphorus), micronutrients (e.g., iron, zinc,), and essential vitamins (e.g., vitamin B12) [36]. In *Fucus vesiculosus* collected from two sites differing in nitrogen availability, polyphenolic concentrations were consistently higher in algae from the low-nitrogen site compared to those from the high-nitrogen site [37]. Similarly, nitrogen enrichment resulted in reduced concentrations of phlorotannins in *F. vesiculosus* [38]. In the red alga *Chondrus crispus*, phosphorus availability does not significantly influence the content of photosynthetic pigments, whereas nitrogen plays a key regulatory role, with increased nitrogen availability enhancing pigment concentrations (chlorophyll a and phycobiliproteins) and nitrogen limitation leading to their reduction [39]. In the same study, the so-called “Neish effect” describes an inverse relationship between nutrient availability and carrageenan content, whereby increasing nitrogen availability decreases carrageenan content, while nitrogen limitation promotes its accumulation. A similar pattern was observed for phosphorus, where low phosphorus availability led to higher carrageenan content, whereas phosphorus enrichment reduced it [39].

#### 2.1.4. Temperature

Besides nutrient availability, other environmental stressors, particularly temperature, strongly influence macroalgal metabolism and the production of BACs. Rising sea surface temperatures associated with global climate change, increasing by more than 0.1 °C per decade since the mid-20th century, have become a major driver of changes in macroalgal physiology, distribution, and biochemical composition [21]. High water temperature triggers white rot disease in *Saccharina japonica*, causing progressive pigment loss and frond whitening [40]. In *Cystoseira crinita*, TPC decreased at 30 °C compared to 25 °C, indicating that higher temperatures suppress phenolic accumulation [41]. In contrast, no direct effect of temperature on phlorotannin levels was observed in *Sargassum patens* [42]. In the brown alga *Laminaria digitata*, elevated temperature accelerated the depletion of the storage polysaccharide laminarin, with reductions of ~90% at 5 °C compared to ~40% at 0 °C over three months, indicating increased metabolic consumption under warmer conditions [43]. Similarly, in red algae *Kappaphycus alvarezii*, increasing temperatures from 28 °C to 40 °C reduced carrageenan and pigment yields [44].

#### 2.1.5. Light Availability

Alongside temperature, light availability (intensity, quality, and UV radiation) strongly influences macroalgal growth and biochemical composition by regulating photosynthesis. Due to spatial and temporal variations in light conditions, macroalgae undergo photoadaptation by adjusting pigment composition, growth, respiration, and metabolism, which affects BAC synthesis [21]. Prolonged exposure to different light wavelengths reduced phlorotannin content in several brown algae species (*Pelvetia canaliculata*, *Fucus vesiculosus*, *Ascophyllum nodosum*, and *Himanthalia elongate*) [45]. Higher photon flux densities inhibited growth and reduced pigment content in red algae *Bostrychia montagnei* and *B calliptera* [46]. Higher irradiance reduced chlorophyll content and changed composition of oxygenated carotenoids in green algae *Codium tomentosum* and *Bryopsis plumosa*.

#### 2.1.6. Salinity

Salinity affects algal osmotic balance and physiology, although tolerance varies among species. Most macroalgae thrive at salinities between 33 and 35 psu [21]. Among Arctic brown algae, some species remained stable across a wide salinity range, some exhibited pigment loss or high mortality under hyposaline conditions and some survived both low and high salinities but showed reduced photosynthetic activity [47]. In brown algae *Ascophyllum nodosum* and *Fucus vesiculosus*, reduced salinity led to a decrease in TPC and altered phenolic composition, with an increased proportion of cell wall-bound phenolics [48]. In *Sargassum muticum*, reduced salinity increased alginate polysaccharide content, while phlorotannin levels slightly increased at moderate salinity (20 ppt). Lower salinities (10–15 ppt) significantly reduced fucoxanthin, whereas only minor changes in chlorophyll a and carotenoids were observed [49].

## 3. Classification and Chemical Characteristics of Macroalgal Bioactive Compounds

Brown, red, and green macroalgae are characterized by a distinct composition of BACs, particularly phenolics, pigments, and polysaccharides, which determine their physiological properties, pigmentation and ecological adaptation to adverse environmental conditions. Following an overview of how environmental factors influence BAC profiles, this chapter examines their classification and chemical characteristics. Understanding these chemical features is essential for interpreting their biological activities, ecological functions, and potential for industrial exploitation. Macroalgal BACs participate in key physiological processes such as photosynthesis, the formation of cell structures and protection from environmental stresses [16], and also show considerable potential for applications in the food, pharmaceutical and cosmetic industries due to their biological activities.

### 3.1. Phenolic Compounds

Phenolic compounds are among the most interesting and biologically significant groups of secondary metabolites in macroalgae, although they constitute only a relatively small proportion of the total chemical composition [50]. Their importance lies not in their abundance, but in the extremely wide range of biological functions they perform. Structurally, phenolic compounds are characterized by one or more aromatic rings with attached hydroxyl (–OH) groups. These chemical features make them highly reactive [51,52]. As discussed previously, these compounds play a crucial role in adaptation to dynamic and stressful marine conditions, including UV radiation, temperature and salinity fluctuations, oxidative stress, and biotic interactions [50]. Among macroalgal phenolics, phlorotannins are particularly notable, being almost exclusively found in brown algae (*Phaeophyceae*) and considered their chemical hallmark [53]. They are polymers of phloroglucinol (1,3,5-trihydroxybenzene) and exhibit remarkable structural diversity. Depending on the type of linkage between monomer units (–C–C–, –C–O–C–, or both), phlorotannins form linear, branched, or polycyclic structures with molecular masses ranging from small oligomers (126 Da) to large macromolecules (650 kDa) [54]. Based on linkage type, they are classified as fucols, phlorethols, fucophlorethols, fuhalols, and the more complex eckols and carmalols, which contain dibenzodioxin rings [55]. Research on eight species of brown macroalgae has shown significantly higher concentrations of phlorotannins (above 2% dry matter (dm)) in samples from the order *Fucales* (up to ten folds higher than those from the order *Laminariales*). Among the seven species from the order Fucales, the highest yields were recorded in *Ascophyllum nodosum* and *Fucus vesiculosus* (up to 5.8% dm) [56]. A similar observation was made in a study on ten different species from eight genera of brown macroalgae (*Cystoseira*, *Sargassum*, *Fucus*, *Halopteris*, *Stypocaulon*, *Cladostephus*, *Padina*, *Saccorhiza*). Significantly higher concentrations of phlorotannins (1.19–26.41 folds higher) were observed in the *Fucus spiralis* sample compared to other species of brown macroalgae studied [57]. In addition to phlorotannins, macroalgae contain other important groups of phenolic compounds, including phenolic acids. Based on their chemical structure, phenolic acids are derivatives of aromatic compounds that contain an aromatic ring (benzene nucleus), one or more hydroxyl (–OH) groups, and a carboxyl group (–COOH) [58]. They are classified as hydroxybenzoic acid derivatives (C6-C1) or hydroxycinnamic acid derivatives (C6-C3). The most common hydroxybenzoic acid derivatives (HBA) in macroalgae are gallic, protocatechuic, *p*-hydroxybenzoic, and vanillic acids [59]. The most common hydroxycinnamic acid derivatives (HCA), in macroalgae are caffeic, ferulic, *p*-coumaric, and sinapic acids [60]. Phenolic acids are more abundant in green and red macroalgae, while phlorotannins are the dominant phenolic compounds in brown algae.

Gallic acid was detected in 16 different macroalgal species, with the highest concentration recorded in the green macroalga *Enteromorpha intestinalis* (17.1 mg/g extract). In addition to gallic acid, higher concentrations of hydroxybenzoic acid (0.3–27.4 mg/g extract) were observed in all samples except those from the order *Fucales*. Other phenolic acids were present in significantly lower concentrations or were absent. Specifically, coumaric acid was detected only in the *Ulva lactuca* sample (0.2 mg/g extract), and ferulic acid only in the *Chondrus crispus* sample (0.1 mg/g extract) [61].

A particularly interesting and biologically active group of phenols is bromophenols. Their chemical and biological properties are determined by the substitution of bromine atoms on the aromatic ring [62] based on their classification as mono-, di-, or tribromophenols [63,64]. Their structural characteristics are further influenced by the position of the substituents [65]. Unlike phlorotannins, bromophenols are present in macroalgae in smaller quantities but exhibit very high biological activity. They are most abundant in red macroalgae, and less frequently found in brown and green macroalgae. In a study of 49 macroalgae species, the lowest yields of total bromophenols were recorded in the green macroalga *Codium fragile* (up to 0.9 ng/g dm), and the highest in the red macroalga *Pterocladiella capillacea* (up to 2590 ng/g dm). Regardless of the macroalga type, only 2,4,6-tribromophenol was present in all investigated macroalgae, while other bromophenol forms were present in 6–62% [66].

Phenolic compounds mainly provide protection against oxidative and environmental stress, supporting the stability and function of the photosynthetic apparatus in macroalgae. In contrast, pigments such as chlorophylls, carotenoids, and phycobiliproteins enable light energy absorption and facilitate the photosynthetic process [67]. These pigments also determine the characteristic color of macroalgae and allow adaptation to different light conditions in the marine environment.

### 3.2. Pigments

Chlorophylls as the primary photosynthetic pigments are found in the highest quantities in green macroalgae. The green macroalga *Ulva lactuca* had a total chlorophyll content of 21.27 mg/g dm, with chlorophyll a as the predominant pigment [68]. Green algae also contain chlorophyll b, which transfers energy to chlorophyll a and facilitates macroalgae adaptation to low-light conditions [69]. Brown algae contain chlorophylls a and c, while red algae mainly contain chlorophyll a and, rarely, chlorophyll d. Chlorophyll d attracts significant interest among researchers because it absorbs in the far-red light spectrum (700–800 nm) [70], allowing red macroalgae to photosynthesize in deep-water environments where red light penetration is minimal. In addition to chlorophylls, the second major group of pigments isolated from macroalgae is carotenoids. These lipophilic tetraterpenes, classified as carotenes and xanthophylls, absorb light in the blue-green region of the spectrum (400–550 nm) and facilitate energy transfer to chlorophylls a and c [71,72]. Unlike carotenes, xanthophylls contain oxygenated functional groups that increase hydrophilic–lipophilic interactions [73] and protect macroalgae from oxidative stress and chlorophylls from photooxidation [74]. β-Carotene is the predominant carotene in macroalgae and has been reported in both green (*Caulerpa racemosa* and *C. taylorii*) and brown species (*Ascophyllum nodosum*, *Bifurcaria bifurcata*, *Fucus spiralis*, *Himanthalia elongata*, *Laminaria ochroleuca*, *Laminaria saccharina*, *Pelvetia canaliculata*, *Sargassum muticum*, and *Undaria pinnatifida*) at concentrations of 0.02–0.04 and 0.01–0.30 mg/g dm, respectively [75,76]. Among the xanthophylls, the most abundant in macroalgae are fucoxanthin and lutein. Fucoxanthin is responsible for the brown-golden color of brown macroalgae, allowing brown algae to photosynthesize even in low light conditions [77]. Concentrations of fucoxanthin in *Undaria pinnatifida* ranged from 3.32 to 4.96 mg/g dm, depending on environmental conditions and harvesting time [78]. Although found in relatively low concentrations in macroalgae, lutein selectively accumulates in the human macula, where it protects the eye from damage caused by ultraviolet and high-energy visible light [79].

Red macroalgae contain a unique group of water-soluble pigments called phycobiliproteins, mainly phycoerythrin and phycocyanin, which are responsible for their characteristic red coloration [80,81]. Generally, regardless of the type of red macroalga, the quantity of phycoerythrin is significantly higher than the concentration of phycocyanin. In *Gracilaria hikkaduwensis*, yields of the pigment phycoerythrin were recorded as 1.73 folds higher [82]. A similar trend of higher phycoerythrin yields was also observed in *Porphyridium cruentum* and *Porphyridium purpureum* samples [83].

### 3.3. Polysaccharides

The main group of BACs in macroalgae is polysaccharides, which are complex carbohydrates composed of numerous monosaccharide units linked by glycosidic bonds. Although polysaccharides are generally classified as primary metabolites, numerous studies have shown that marine polysaccharides, including fucoidan, laminarin, alginate, carrageenan, and ulvan, exhibit a wide range of biological activities, such as antioxidant, anti-inflammatory, immunomodulatory, antiviral, and anticancer effects. Thus, despite their primary metabolic origin, these compounds are increasingly recognized as BACs because of their ability to elicit specific physiological responses and provide health benefits beyond their fundamental biological roles. Consequently, marine polysaccharides are included in this review as BACs on the basis of their demonstrated functional properties and significant potential for applications in food, nutraceutical, and pharmaceutical products. Polysaccharides in macroalgae have a dual role: structural polysaccharides contribute to the formation of the cell wall, while storage polysaccharides serve as an energy reserve [84]. Their structure and composition vary depending on the type of macroalga. Among the most significant polysaccharides present in the cell walls of green macroalgae are ulvan, cellulose, mannan and xylan [85]. These polysaccharides contribute to the mechanical stability of cells, protect against external influences, and participate in numerous biological processes. A particularly important polysaccharide in most green macroalgae is ulvan, which is present in the cell walls at up to 36% [86]. Its concentration depends not only on the species but also on the harvesting time. In the same species of green macroalgae, *Ulva lactuca*, during the period of intensive growth, the ulvan content in the cell walls of young macroalgae was observed to be lower. The polysaccharide ulvan is a sulphated heteropolysaccharide characteristic of order *Ulvaceae*, with a complex chemical structure composed of different monosaccharide units (45% rhamnose, 9.6% xylose, 9.6% glucuronic acid, and 5% iduronic acid), to which a sulphate group is covalently bound [87]. The presence of sulphate groups gives this polysaccharide distinctive physicochemical properties, such as increased hydrophilicity, the ability to bind water, and interaction with various ions. Its ability to form gels and films makes ulvan suitable for applications in the food industry, pharmacy, and biomedicine, particularly in the development of biomaterials and controlled drug release systems [88,89,90]. Along with ulvan, cellulose is also an important structural component of the cell walls of green macroalgae. [91], forming microfibrils that create a solid, organized network providing mechanical strength and resistance to cells, enabling them to maintain their shape. Cellulose microfibrils are often embedded in a matrix of other polysaccharides, which further increases the stability of the cell wall. Compared with terrestrial plants, cellulose extraction from macroalgae is more energy-efficient and environmentally friendly because macroalgae do not contain lignin [92]. Similar to ulvan, macroalgae from the order *Ulvaceae* contain significant amounts of cellulose, which can reach up to 855 mg/g dm [93]. The cell walls of green macroalgae also contain various hemicellulose polysaccharides, among which β-mannan and β-xylan are the most abundant [94]. These polysaccharides play a key role in linking cellulose microfibrils and forming a complex three-dimensional network within the cell wall [95]. The combination of cellulose, ulvan, and hemicelluloses provides green macroalgal cell walls with mechanical strength, flexibility, and resistance to environmental stress, such as waves and water currents, ensuring efficient exchange of substances between the cell and its surroundings [90]. On the other hand, brown macroalgae are characterized by the presence of the polysaccharide’s alginates, fucoidans, and laminarin [96]. Alginates are the most abundant polysaccharides in brown macroalgae, comprising up to 40% of the *Sargassum* dry matter). They consist of β-D-mannuronic acid and α-L-guluronic acid, and the ratio and arrangement of these acids determine their physicochemical properties, particularly their gel-forming ability [97,98]. Alginates with a higher proportion of guluronic acid form firm gels (e.g., with Ca^2+^ ions), while those with a higher proportion of mannuronic acid form softer, more elastic gels [99]. For this reason, alginates are widely used as thickeners and stabilizers in the food industry. Unlike alginates, which provide macroalgae with strength and elasticity in the cell wall, laminarin serves as the primary storage polysaccharide, and fucoidan has mainly structural and bioactive roles [100]. Fucoidans are complex sulphated polysaccharides rich in L-fucose monomer units, whose structure may include various glycosidic bonds and additional monosaccharides [101]. Due to the sulphate group, fucoidan is chemically similar to the animal polysaccharide heparin, so its anticoagulant effect has been the subject of recent studies. Notably strong anticoagulant activity was observed in fucoidans isolated from *Laminaria saccharina*, *L. digitata*, *Fucus serratus*, *F. distichus*, and *F. evanescens* [102]. Laminarin is another abundant reserve polysaccharide in brown macroalgae, consisting mainly of β-1,3-linked glucose units with occasional β-1,6 branches and serving as an energy source in algal cells [103].

Unlike brown and green macroalgae, red macroalgae contain the characteristic polysaccharides agar and carrageenan, which belong to the group of sulphated galactans [104]. Agar consists of two main components: agarose and agaropectin. Agarose, a neutral linear polysaccharide that constitutes about 70% of agar, is composed of repeating units of β-D-galactose and 3,6-anhydro-L-galactose and is responsible for the gel-forming properties of agar. In contrast, branched agaropectin contains additional groups such as sulphate esters, is more highly charged, and contributes less to gelation [104,105]. Because it can form solid and stable gels, agar is widely used in the food industry and microbiology as a medium for cultivating microorganisms.

Carrageenans are also galactan polysaccharides, composed of repeating units of D-galactose and 3,6-anhydro-D-galactose. The most important carrageenans, κ-, ι-, and λ-carrageenans, differ in the degree and arrangement of sulphate ester groups, which significantly affects their physicochemical properties [106]. In κ-carrageenan, one sulphate ester group is present per disaccharide unit, allowing the formation of solid and relatively rigid gels, especially in the presence of potassium ions. ι-carrageenan contains one additional sulphate ester group per disaccharide, forming softer, elastic gels, most often stabilized by Ca^2+^ ions. In contrast, λ-carrageenan has three sulphate ester groups per disaccharide unit, making it highly soluble in water and unable to form gels, but increasing the viscosity of solutions [107]. Research on macroalgae from the genera *Chondrus*, *Euchema*, *Furcellaria*, *Fucus*, *Gigartina*, *Hypnea*, *Iridae*, and *Kappaphycus* has determined the proportion of sulphate ester groups present: 25–30% in κ-carrageenan, 28–30% in ι-carrageenan, and 32–39% in λ-carrageenan [108]. The degree of sulphation of carrageenan directly affects its electrical charge, interactions with ions, and ability to form a three-dimensional network in water. Because of these useful technological properties, carrageenans are widely used in the food industry as thickeners, stabilizers, and gelling agents in products such as dairy desserts, sauces, and confectionery.

## 4. Advanced Green Extraction Techniques for Recovery of Macroalgal Bioactive Compounds

The growing interest in marine macroalgae as a sustainable source of BACs for food, nutraceutical, and pharmaceutical applications has stimulated the development of extraction techniques capable of improving both recovery efficiency and product quality. Conventional extraction (CE) methods, including maceration, reflux extraction, and Soxhlet extraction, have usually been used for recovering macroalgal metabolites. However, these methods are often associated with longer extraction times and temperatures, high energy consumption, and large volumes of organic solvents, which may cause the structural changes and degradation of thermolabile compounds. These limitations are especially problematic in macroalgae, where BACs are often embedded within complex, polysaccharide-rich cell walls that hinder mass transfer and reduce extraction efficiency. Furthermore, prolonged exposure to heat and oxygen can promote oxidation, isomerization, and degradation of thermolabile BACs, including phenolics, pigments, and certain polysaccharides, resulting in reduced antioxidant and anti-inflammatory activities in the extracts obtained. Therefore, efficient extraction of BACs from marine macroalgae is one of the most critical steps in developing algal-derived ingredients. The effectiveness of any extraction process ultimately determines not only the extraction yield but also the chemical composition, purity, stability, and biological activity of the resulting extracts, directly influencing their commercial value and industrial applicability [109]. To address these limitations, a range of advanced extraction techniques, commonly referred to as green extraction methods, has been developed. Among the most extensively studied approaches for macroalgal processing are microwave-assisted extraction (MAE), ultrasound-assisted extraction (UAE), and supercritical fluid extraction (SFE) [110]. Although these techniques differ in their mechanisms, they share several common objectives: improving mass transfer, enhancing the accessibility of target compounds within algal tissues, reducing extraction time and solvent use, minimizing thermal degradation, and increasing overall process sustainability [111].

As a result, these techniques are increasingly regarded as key tools for the efficient valorization of macroalgal biomass within circular bioeconomy and green biotechnology frameworks [109]. A fundamental advantage of these technologies is their ability to enhance mass transfer from macroalgal tissue to the extraction solvent. Improved penetration of the extraction solvent into cellular structures facilitates the release of intracellular metabolites that are otherwise difficult to recover using CEs. Consequently, green extraction techniques often produce extracts with higher concentrations of BACs and greater AOA than those obtained by CE [111,112,113,114,115]. This characteristic is particularly important for antioxidant and anti-inflammatory applications, where biological efficacy is closely linked to the concentration, structural integrity, and synergistic interactions of multiple compounds present in the extract.

Phenolic compounds are among the most extensively studied macroalgal metabolites recovered through advanced extraction technologies. Brown macroalgae, in particular, contain phlorotannins, a unique class of marine polyphenols that has demonstrated strong antioxidant, anti-inflammatory, antidiabetic, neuroprotective, and anticancer activities. Numerous studies have reported significantly improved recovery of phenolic compounds using MAE, UAE, and SFE compared with CE [113,116,117]. In addition to increasing extraction yield, these techniques often better preserve phenolics that are susceptible to degradation during prolonged thermal treatment. As a result, optimized extraction conditions frequently yield extracts with enhanced AOA and stronger inhibition of key enzymes involved in metabolic disorders, including α-amylase, α-glucosidase, pancreatic lipase, and tyrosinase [116,117]. Natural deep eutectic solvents (NADES) have recently emerged as a promising class of green solvents for extracting macroalgal BACs, owing to their low toxicity, biodegradability, and tunable physicochemical properties. In particular, NADES have been successfully used to extract phlorotannins from *Fucus vesiculosus* and *Ascophyllum nodosum*, demonstrating high extraction efficiency comparable to that obtained with methanol and ethanol [118,119]. A fast and green NADES-UAE procedure was developed for the extraction of phlorotannins in Arctic *Fucus vesiculosus* and 32 individual phlorotannins (one trimer, two tetramers, six pentamers, four hexamers, six heptamers, six octamers, and seven nonamers) were identified in the extracts by HPLC-HRMS and MS/MS techniques [120].

Beyond phenolics, advanced extraction techniques have proven highly effective in recovering photosynthetic pigments, including fucoxanthin, chlorophylls, and carotenoids [112,121,122,123,124,125]. These compounds have attracted increasing attention due to their antioxidant and anti-inflammatory properties, as well as their potential roles in preventing obesity, metabolic syndrome, and cancer. Fucoxanthin is a characteristic carotenoid of brown macroalgae and is particularly notable for its broad spectrum of biological activities. It is abundant in species such as *Sargassum*, *Undaria*, *Fucus*, *Laminaria*, and *Dictyota* [77,126]. Studies have shown that optimized extraction procedures can significantly increase fucoxanthin recovery while maintaining pigment stability and bioactivity [125,127,128]. Importantly, extraction conditions influence not only pigment yield but also their specific composition. Recent evidence suggests that fucoxanthin extracts enriched in Z-isomers may have improved bioavailability and enhanced biological activities compared with all-E isomers, highlighting the importance of extraction conditions in determining the functional value of the final product [125]. These findings demonstrate that extraction should not be viewed merely as a recovery process but as a critical step that can influence the biological performance of the extracted compounds.

Polysaccharides represent another major group of macroalgal BACs that benefit substantially from advanced extraction approaches. Sulphated polysaccharides, including fucoidan, carrageenan, ulvan, and agar-derived oligosaccharides, have attracted considerable scientific interest because of their diverse biological activities, such as antioxidant, anti-inflammatory, immunomodulatory, anticoagulant, antiviral, and prebiotic effects [113,114,115,129,130,131]. Unlike low-molecular-weight metabolites, the biological activity of polysaccharides is strongly influenced by structural characteristics such as molecular weight, degree of sulphation, monosaccharide composition, and glycosidic linkage patterns. Therefore, extraction techniques must not only maximize recovery yield but also preserve critical structural features responsible for biological properties. Numerous studies have shown that optimized green extraction techniques can improve both extraction efficiency and the functional properties of polysaccharides, including water-holding capacity, oil-holding capacity, emulsification potential, and enzyme inhibitory activity [115,129,132]. A recent study comparing dynamic maceration and UAE for fucoidan recovery from four Arctic brown algae showed that UAE significantly increased fucoidan and uronic acid yields, whereas dynamic maceration produced fucoidans with higher phlorotannin content and antioxidant activity, particularly from Ascophyllum nodosum and *Fucus vesiculosus* [133].

These characteristics are particularly valuable for the development of functional foods and nutraceutical formulations. An increasingly important concept in macroalgal processing is the recognition that extraction techniques should not be evaluated solely by extraction yield. Instead, the quality and functionality of recovered compounds are becoming equally important criteria. High extraction yields have limited practical significance if extraction conditions cause degradation of sensitive metabolites or reduce their biological activity. Therefore, current research increasingly focuses on optimizing extraction conditions to achieve the best balance between yield, selectivity, structural preservation, and biological efficacy [113,114,130]. This shift reflects the growing demand for high-value functional ingredients rather than bulk extracts with poorly defined composition. Another important consideration is the integration of extraction techniques into macroalgal biorefinery systems. Modern biorefinery concepts aim to utilize the entire macroalgal biomass through sequential recovery of multiple product streams, maximizing economic value while minimizing waste. Within these frameworks, advanced extraction techniques play a central role by enabling selective fractionation of biomass into distinct groups of compounds, including pigments, phenolics, lipids, proteins, and polysaccharides [113,134,135].

More recently, the simultaneous extraction of hydrophilic phlorotannins and ascorbic acid, along with lipophilic fucoxanthin, from *Fucus vesiculosus* using NADES has been reported, demonstrating the potential of these solvents for integrated macroalgal biorefinery approaches and the efficient recovery of compounds with different polarities [136]. In addition, the favorable safety profile of NADES and their low co-extraction of potentially toxic metals further support their use as safe, sustainable extraction media for food, cosmetic, and pharmaceutical products [137].

For example, supercritical CO_2_ extraction is often used as an initial defatting step to remove lipophilic compounds before subsequent recovery of polysaccharides and other polar compounds [127,135]. Such integrated processing strategies contribute significantly to the economic and environmental sustainability of macroalgal valorization. Despite their considerable advantages, the industrial implementation of MAE, UAE, and SFE remains associated with several common challenges. One of the most significant barriers is scale-up. Extraction efficiencies achieved under laboratory conditions are often difficult to reproduce at industrial scale because of limitations in heat and mass transfer, energy distribution, reactor geometry, and process control. Maintaining homogeneous extraction conditions throughout large processing volumes is particularly challenging and may result in variability in extract composition and quality. Economic considerations also represent an important constraint. Advanced extraction approaches generally require substantially higher capital investment than CE equipment. Furthermore, process optimization often demands sophisticated monitoring systems and highly trained personnel, increasing operational costs [111,138,139,140,141,142]. Regulatory requirements present an additional challenge, particularly for food and nutraceutical applications where solvent residues, product safety, traceability, and environmental sustainability must be rigorously controlled. Future development of macroalgal extraction techniques will likely focus on improving process integration, automation, energy efficiency, and sustainability [113,134,135]. The combination of advanced extraction methods with emerging approaches such as enzyme-assisted extraction, deep eutectic solvents, membrane separation technologies, and artificial intelligence-driven process optimization may further enhance recovery efficiency and product quality [120,141,143]. At the same time, life-cycle assessment and techno-economic analyses will become increasingly important for evaluating the overall sustainability of extraction processes and supporting industrial decision-making [142]. Overall, advanced extraction technologies have transformed the recovery of BACs from marine macroalgae by enabling higher extraction efficiency, improved preservation of bioactivity, and greater process sustainability than conventional approaches. Their continued development will be essential for fully exploiting the potential of macroalgae as a renewable source of BACs for functional foods and nutraceuticals.

## 5. Mechanisms of Antioxidant and Anti-Inflammatory Activity of Macroalgal Bioactive Compounds

Increasingly, scientific interest has focused on understanding the structural features of macroalgal BACs that underlie their antioxidant and anti-inflammatory activities, as better knowledge of their mechanisms of action enables more targeted applications in the food and nutraceutical industries. Phenolic compounds, pigments and polysaccharides derived from macroalgae have been widely reported to exhibit significant biological activities, largely depending on their molecular structure, degree of polymerization, and functional groups. These structural characteristics influence their ability to interact with biological systems through multiple mechanisms such as radical scavenging, metal ion chelation, hydrogen bonding, and modulation of key inflammatory pathways [144,145]. Therefore, the relationship between structure and bioactivity is essential for understanding the physiological relevance of macroalgal compounds and their value as natural agents for promoting health and mitigating inflammation-related disorders.

### 5.1. Oxidative Stress and Antioxidant Mechanisms of Macroalgal Bioactive Compounds

Oxidative stress is defined as an imbalance between oxidants and antioxidants in favor of the oxidants, leading to a disruption of redox signaling and control and/or molecular damage [146]. The intracellular accumulation of reactive oxygen species (ROS), reactive nitrogen species (RNS) and other free radicals can initiate harmful chain reactions in the body that damage cell membranes, inhibit key enzyme activity, disrupt normal cellular processes and division, interfere with energy production and damage cellular DNA. Consequently, oxidative stress is closely associated with the development and progression of numerous chronic diseases, including cardiovascular disorders, diabetes, inflammation, neurodegenerative diseases and cancer [144,147,148,149]. Besides oxidative stress, inflammation also plays an important role in the pathogenesis of these diseases, as oxidative stress and inflammation are complex, interconnected pathophysiological processes. Specifically, oxidative stress can initiate and prolong inflammation through the activation of redox-sensitive signaling pathways, while inflammatory responses stimulate excessive production of ROS and RNS, further amplifying oxidative stress [150,151,152]. ROS, such as superoxide anion (O_2_^•−^), hydrogen peroxide (H_2_O_2_), singlet oxygen (^1^O_2_) and hydroxyl radical (OH•), are highly reactive and unstable species that are by-products of cellular metabolic processes [153]. They can be generated in the mitochondrion through the electron transport chain, produced by enzymes such as NADPH oxidase and xanthine oxidase, or formed in response to environmental factors, including smoke, radiation, excessive drug intake and improper dietary habits [144,149,153]. To protect the organism from the oxidation of biologically important molecules and to repair oxidative damage, the body produces various antioxidant compounds that neutralize free radicals and reduce their harmful effects [144,154]. This complex antioxidant defense system is supported by endogenous enzymatic and non-enzymatic antioxidants [155]. The endogenous enzymatic system includes key enzymes such as superoxide dismutase (SOD), one of the most potent antioxidant enzymes, which catalyzes the conversion of superoxide anion into hydrogen peroxide; catalase (CAT), which decomposes hydrogen peroxide into water and oxygen; and glutathione peroxidase (GPx), which reduces hydrogen peroxide and lipid hydroperoxides using glutathione as a cofactor [144,155,156]. However, these enzymatic systems are complemented by exogenous antioxidants which can act through several mechanisms, including delaying or inhibiting the production of free radicals, scavenging reactive species, interrupting radical chain reactions, enhancing the activity of other chain-breaking antioxidants through synergistic effects, exerting reducing activity, chelating metal ions or inhibiting specific oxidative enzymes [157].

Among these exogenous antioxidants, phenolic compounds exhibit radical-scavenging activity through several complementary mechanisms, including hydrogen atom transfer (HAT), electron transfer (ET), and sequential proton loss electron transfer (SPLET). In the HAT mechanism, phenolics donate a hydrogen atom from their hydroxyl group to neutralize radical species, and the stability of the resulting phenoxyl radical determines their overall radical-scavenging efficiency. In the ET mechanism, phenolic compounds donate a single electron to a free radical, forming phenolic radicals stabilized through resonance. This process depends on the ionization potential of the molecule [158]. The SPLET mechanism involves the initial deprotonation of the phenolic hydroxyl group to form a phenolate anion, which then donates an electron to the radical species, producing a more stable product. In addition to these pathways, certain phenolic antioxidants can chelate transition metals such as iron (Fe^2+^, Fe^3+^) and copper (Cu^2+^, Cu^+^), thereby preventing their participation in Fenton-type reactions that generate highly reactive radicals [159]. Although phenolic compounds are among the most extensively studied macroalgal antioxidants, other classes of BACs such as carotenoids, chlorophylls and phycobiliproteins also contribute significantly to antioxidant defense.

Carotenoids such as astaxanthin, fucoxanthin, and zeaxanthin are among the most efficient physical quenchers of ROS, such as singlet oxygen (^1^O_2_), both in vitro and in vivo [160]. Their AOA involves several free radical-scavenging mechanisms, primarily electron transfer, formation of radical adducts, and hydrogen atom transfer, the latter resulting in the formation of a neutral carotenoid radical [160]. In addition, the AOA of carotenoids may also be mediated through metal chelation, which helps prevent the formation of highly reactive radical species [144].

Polysaccharides such as carrageenans, laminarin, and various sulphated polysaccharides act as antioxidants by directly scavenging free radicals and chelating metal ions [144]. They can also indirectly influence endogenous antioxidant enzymes (SOD, CAT, GPx) by affecting signaling pathways, including the Kelch-like ECH-associated protein 1 (Keap1)—nuclear factor erythroid 2-related factor 2 (Nrf2)—antioxidant response elements (ARE) pathway [161]. Several studies have shown that this pathway plays a crucial role in protecting cells against endogenous and exogenous oxidative stress, inflammation, and xenobiotic-induced damage, and is therefore considered a pharmacological target [162]. Polysaccharides may inhibit oxidases, such as inducible nitric oxide synthase (iNOS), which produce high levels of nitric oxide (NO) during inflammatory responses. NO can react with ROS to generate RNS, thereby contributing to oxidative and nitrosamine stress [161].

To evaluate the antioxidant potential of macroalgal BACs, several in vitro assays are commonly employed. The most widely used methods include DPPH, ABTS, FRAP, and ORAC assays. The DPPH assays assess radical-scavenging activity primarily through single electron transfer mechanisms, ABTS through balancing electron or hydrogen atom donation, while the FRAP assay measures reducing power specifically based on the electron-donating capacity of antioxidants [163]. ORAC, on the other hand, measures the ability of antioxidants to neutralize peroxyl radicals, which are important reactive species in biological systems. In addition to ORAC, superoxide (O2•)- and nitric oxide (NO)-scavenging assays evaluate the ability of the extracts to neutralize biologically relevant ROS and RNS. The combination of AOA assays provides a complementary evaluation of the in vitro AOA of macroalgal extracts, as each assay reflects a different antioxidant mechanism. A number of studies have confirmed the potent antioxidant effect of methanolic, ethanolic, and aqueous extracts of various algal species [164,165,166,167]. According to Shibata et al. [168], the phlorotannins, isolated from the brown algae *Eisenia bicyclis*, *Ecklonia cava* and *Ecklonia kurome*, showed significant radical-scavenging activities against the superoxide anion (EC50%: 6.5– 8.4 μM) and DPPH (EC50%: 12–26 μM), and phlorotannins were more effective than ascorbic acid and α-tocopherol. Among several brown macroalgae studied, *Ascophyllum nodosum* showed the highest AOA, which was positively correlated with its phlorotannin concentration [169]. Extracts from *Sargassum filipendula* demonstrated strong AOA and inhibited diene conjugate formation and thiobarbituric acid reactive substances [164]. They also dose-dependently inhibited collagenase and elastase, indicating potential antioxidant and anti-aging applications. Comparing phenolic extracts of *Ascophyllum nodosum*, *Fucus vesiculosus* and *Bifurcaria bifurcata*, the highest ORAC, DPPH and FRAP antioxidant activities, as well as the strongest correlation between AOA and TPC, were observed in the *B. bifurcata* extract [170]. Similarly, recent studies on Arctic populations of Ascophyllum nodosum, *Fucus vesiculosus*, *F. spiralis*, and *F. distichus* have confirmed their strong antioxidant and antiradical activities, largely associated with their elevated phlorotannin contents [171].

Duan et al. [172] evaluated the DPPH AOA of bromophenols isolated from red alga *Symphyocladia latiuscula* and confirmed their potent AOA, with IC_50_ values ranging from 8.1 to 24.7 μM. In the green alga *Caulerpa racemosa*, the carotenoid extract showed good DPPH and ABTS AOA [173]. Similarly, fucoxanthin isolated from the brown alga *Himanthalia elongata* exhibited notable DPPH (EC_50_ = 12.9 μg/mL) and FRAP (15.2 μg Trolox equivalents) antioxidant activities, although its FRAP antioxidant power was lower than that of commercial fucoxanthin [174]. An overview of recent research on antioxidant effects of macroalgae is presented in Table 1.

### 5.2. Inflammation and Anti-Inflammatory Mechanisms of Macroalgal Bioactive Compounds

The close relationship between oxidative stress and inflammation has made the anti-inflammatory activity of macroalgal BACs an important focus of recent research. Inflammation is a complex cellular and molecular response triggered by injury, irritation, infection, or other pathological states and usually begins with the activation of immune cells (neutrophils and macrophages), which releases various inflammatory mediators [175,176]. Among the most important inflammatory mediators are prostaglandin E_2_ (PGE_2_), nitric oxide (NO), pro-inflammatory enzymes (inducible nitric oxide synthase (iNOS), cyclooxygenase-2 (COX-2)), and pro-inflammatory cytokines (tumor necrosis factor-α (TNF-α), interleukin-1β (IL-1β), and interleukin-6 (IL-6)). In addition, signaling pathways such as nuclear factor kappa-B (NF-κB) and mitogen-activated protein kinase (MAPK) coordinate various physiological and pathological processes associated with inflammation through regulation of inflammatory gene expression and activation of immune cells [9].

Many BACs from macroalgae exert anti-inflammatory effects through multiple complementary mechanisms. They influence the production of pro-inflammatory cytokines, including IL-1β, IL-6, TNF-α, monocyte chemoattractant protein-1 (MCP-1), and macrophage inflammatory protein-1α (MIP-1α). In addition, macroalgal BACs can inhibit key inflammatory enzymes, such as iNOS, thereby lowering NO and COX-2 production, resulting in decreased levels of prostaglandin PGE_2_ [145] which contributes to attenuation of the inflammatory response. These effects are closely associated with inhibition of intracellular signaling pathways which suppresses the transcription of pro-inflammatory mediators and limits the activation of immune cells. Furthermore, some BACs enhance cellular antioxidant defenses by activating the nuclear factor erythroid 2–related factor 2 (Nrf2) pathway and inducing heme oxygenase-1 (HO-1) expression, thereby reducing oxidative stress and inflammation. The anti-inflammatory effects of macroalgal polyphenols, phlorotannins, carotenoids, and sulphated polysaccharides have been evaluated in various experimental models such as in vitro cell-based assays, biochemical analyses of inflammatory markers, molecular studies of intracellular signaling pathways, and in vivo models of inflammation. Numerous experimental studies have shown that specific macroalgal BACs suppress NF-κB and MAPK signaling, downregulate the expression of iNOS, COX-2, and pro-inflammatory cytokines, and enhance antioxidant defenses via the Nrf2 pathway, thereby attenuating excessive inflammatory responses [165,177,178,179,180]. For example, Park et al. [181] showed that fucoidan inhibited excessive production of NO and PGE2 in LPS-stimulated BV2 microglia, and reduced the production of iNOS, COX-2, MCP-1, and pro-inflammatory cytokines, such as IL-1β and TNF-α. It also inhibited NF-κB activation and reduced extracellular signal-regulated kinase (ERK), c-Jun N-terminal kinase (JNK), p38 MAPK and protein kinase B (AKT) pathways. In addition, fucoidan isolated from *Fucus vesiculosus* exhibited significant inhibition of COX-2 activity (IC_50_ = 4.3 μg mL^−1^), with greater selectivity than the synthetic anti-inflammatory drug indomethacin, as well as concentration-dependent inhibition of hyaluronidase (IC_50_ = 2.9 μg mL^−1^) and attenuation of lipopolysaccharide-induced p38 MAPK expression, further highlighting the multifaceted anti-inflammatory mechanisms of macroalgal sulfated polysaccharides [182]. Moreover, fucoidans from five species of brown algae were shown, for the first time, to exert anti-inflammatory effects through concentration-dependent inhibition of protein denaturation and stabilization of human erythrocyte membrane corpuscles, with these activities strongly associated with fucose content and moderately associated with sulfate content [183]. The anti-inflammatory potential of fucoidan from *Fucus vesiculosus* L. was confirmed in vivo. A fucoidan-based cream dose-dependently inhibited carrageenan-induced edema and alleviated mechanical allodynia in rats, with the highest dose exhibiting efficacy comparable to diclofenac gel, highlighting the potential of fucoidan for topical anti-inflammatory formulations [184].

Furthermore, phlorotannin extract from the brown alga *Ecklonia cava* showed pronounced anti-inflammatory effects by reducing serum levels of NO, PGE_2_, and HMGB-1, decreasing the expression of iNOS, COX-2, TNF-α, IL-6, and HMGB-1, suppressing the NIK/TAK1/IKK/IκB/NF-κB signaling pathway, and increasing the expression of the antioxidant factors Nrf2 and HO-1 in a mouse model of LPS-induced septic shock [185]. An aqueous extract derived from the alga *Sargassum siliquastrum* inhibited the production of pro-inflammatory cytokines, prostaglandin E2, and NO in an LPS-stimulated RAW 264.7 macrophage model. Furthermore, it exerted protective effects against D-galactose-induced aging by suppressing the NF-κB/AP-1 and MAPK signaling pathways in mice [186]. Farrugia et al. [187] investigated the effects of astaxanthin on the mRNA and protein expression of pro-inflammatory and antioxidant genes, as well as on the accumulation of ROS, in RAW 264.7 macrophages and mouse bone marrow-derived macrophages. Astaxanthin significantly reduced the mRNA expression of IL-6 and IL-1β by inhibiting the nuclear translocation of NF-κB p65 and attenuating LPS-induced ROS production. Choi et al. [188] reported that the bromophenol isolated from the red alga *Polysiphonia morrowii*, and exerted potent anti-inflammatory effects in LPS-induced macrophage cells by reducing the production of inflammatory mediators such as NO, PGE_2_, iNOS, COX-2, and the pro-inflammatory cytokines TNF-α, IL-1β and IL-6. BBDE affected signal transduction and ROS production by selectively inhibiting ERK phosphorylation and suppressing LPS-induced ROS generation in RAW 264.7 macrophages. An overview of recent research in the field of anti-inflammatory effects of macroalgae is given in Table 1. The methods used provide a comprehensive framework for characterizing the mechanisms by which macroalgal BACs modulate inflammation and for evaluating their therapeutic potential. The available evidence generally indicates that macroalgal BACs have significant anti-inflammatory potential through the modulation of multiple molecular targets and signaling pathways, including neutralizing free radicals, protecting lipids and proteins from oxidation, and modulating inflammatory processes at the cellular level. Their combined antioxidant and anti-inflammatory properties support their potential use as functional ingredients, nutraceuticals, and therapeutic agents. However, despite extensive in vitro and in vivo evidence, further research is needed to optimize extraction and standardization procedures, clarify molecular mechanisms of action, and establish efficacy and safety through well-designed clinical studies. Addressing these challenges is essential to fully realize the biomedical and biotechnological potential of macroalgal BACs.

**Table 1:** The antioxidant and anti-inflammatory effects of macroalgal extracts.

BAC Extract	Algae	Study Model	Dose/Treatment	AOA/Anti-Inflammatory Activity	References
Extractable and non-extractable polyphenols	*Laminaria japonica* (B) *Ulva lactuca* (G) *Porphyra tenera* (R)	DPPH and ABTS assays, in vitro: LPS-stimulated RAW 264.7 Macrophages; HCT116 human colon cancer cells	Tested at multiple concentrations; non-cytotoxic up to 200 μg/mL	High DPPH and ABTS AOA-s of extractable polyphenols from *L. japonica* and *U. lactuca*. Reduced NO production in activated macrophages and upregulated the antioxidant enzymes HO-1 and NQO-1. Exerted strong inhibitory effects in activated macrophages by suppressing the proinflammatory cytokines (IL-1, IL-6, TNF-α). Downregulated iNOS and COX-2 expression in activated macrophages. Decreased the proliferation of HCT116 cells by inducing cell cycle arrest and apoptosis.	[177]
Polyphenolic extracts	*Laminaria japonica* (B) *Undaria pinnatifida* (B) *Sargassum fusiforme* (B) *Ascophyllum nodosum* (B)	in vitro: DPPH assay, H_2_O_2_- induced oxidative stress in HaCaT cells	5 μg/mL polyphenolic extract; 1 mM H_2_O_2_ (1 h), followed by extract treatment for 23 h	Highest AOA of *A. nodosum* extract which correlated with TPC and protected HaCaT cells against H_2_O_2_-induced oxidative damage.	[169]
Polyphenol extract Fucoidan extract	*Sargassum filipendula* (B)	in vitro: DPPH, ABTS, O_2_•^−^, and •OH radical scavenging assays; methyl linoleate lipid peroxidation model; collagenase and elastase inhibition assays	concentration-dependent assays, collagenase/elastase inhibition IC_50_ = 0.04–1.61 mg/mL; epigallocatechin gallate as positive control.	High free radical-scavenging activity. Inhibition of lipid peroxidation, conjugated dienes and TBARS formation. Dose-dependent inhibition of collagenase and elastase activities.	[164]
Phenolic extracts	*Dictyota dichotoma* (B) *Padina pavonica* (B)	in vitro: ORAC, FRAP and DPPH assays	Multiple concentrations	Good reducing capacity and ORAC AOA (↑*D. Dichotoma*). Low DPPH AOA except ethanol extracts of *P. pavonica*	[167]
Polysaccharide-rich extract	*Ulva sp*. (G) *Laminaria ochroleuca* (B) *Chondrus crispus* (R)	In vitro: ABTS, DPPH, ORAC, **·**NO, O_2_^•−^ scavenging assays. COX inhibition and HRBC membrane stabilization assays	Multiple concentrations	Highest AOA of *L. ochroleuca* ethanol–water extract and anti-inflammatory potential through inhibition of COX-2 activity.	[189]
Fucoidan extract	*Undaria pinnatifida* (B) *Fucus vesiculosus* (B) *Macrocystis pyrifera* (B) *Ascophyllum nodosum* (B) *Laminaria japonica* (B)	In vitro: LPS-stimulated PBMC and THP-1 macrophages	10–200 μg/mL; LPS-stimulated PBMCs (200 ng/mL) and THP-1 macrophages (1 μg/mL)	Reduced cytokine production in LPS-stimulated PBMCs and human THP-1 cells (dose-dependent). The lowest molecular-weight subfractions showed maximal anti-inflammatory effects at low concentrations.	[179]
Laminarin (commercial)	*Laminaria digitata* (B)	in vitro: HDFa and NHEK skin cells	1–500 μg/mL; antioxidant effects from 1 to 10 μg/mL; no cytotoxicity up to 500 μg/mL exposed to H_2_O_2_ or UVA	Reduced the production of ROS. Significantly reduced basal and induced ROS levels under oxidative stress conditions (H_2_O_2_, UVA radiation). Reduced mitochondrial activity for NHEK cells and for HDFa cells. Modulated cell surface glycosylation and cytokine secretion from skin cells.	[190]
Sulfated iota-carrageenan	*Solieria filiformis* (R)	In vivo: Naproxen-induced gastrointestinal injury in mice	10–90 mg/kg (optimal effect at 30 mg/kg)	Preserved gastrointestinal antioxidant defense and prevented lipid peroxidation, reduced non-protein sulfhydryl group and malondialdehyde concentrations induced by naproxen. Mitigated naproxen-induced gastrointestinal inflammation, reduced MPO activity, TNF-α, and IL-1β.	[180]
Carotenoid extracts	*Caulerpa racemosa* (G)	in vitro: DPPH, ABTS and AMPK anti-inflammatory assays; α-glucosidase and α-amylase inhibition assays; cytotoxicity assay on normal cell	Multiple concentrations (dose-dependent; non-cytotoxic to normal cells)	High DPPH and ABTS AOA. Inhibition of α-glucosidase, α-amylase, the TNF-α and mTOR. Upregulation of AMPK.	[173]
Astaxanthin, and xanthophyll extracts	*Pyropia yezoensis* (R)	in vitro: IFN-γ (10 ng/mL)/TNF-α-(10 ng/mL) stimulated HaCaT cell line	Multiple concentrations (non-cytotoxic)	Reduced production of inflammatory-mediated chemokines in IFN-γ/TNF-α-induced. HaCaT cells through the inactivation of the NF-κB and MAPK pathway. The effect on the ERK and other MAPKs was related to the suppression of TARC and MDC production by blocking NF-κB activation in HaCaT cells.	[178]
Ethanol and acetone extracts of pigments	*Halopteris scoparia* (B) *Sargassum hornschuchii* (B) *Corallina elongate* (R)	in vitro: DPPH assay	UAE extracts obtained under different extraction conditions (96% ethanol or 80% acetone; 30–50 °C; 10–30 min)	High DPPH AOA and correlation between the total carotenoids content and chlorophyll a and b.	[122]
Phycoerythrin extract	*Kappaphycus alvarezii* (R)	in vitro antioxidant assays: total antioxidant, H_2_O_2_ scavenging, reducing power, DPPH, and ABTS	Multiple concentrations	High AOA (total antioxidant capacity, hydrogen peroxide scavenging, reducing power, DPPH, ABTS)	[191]

Abbreviations: B—brown algae; G—green algae; R—red algae; AOA—antioxidant activity-; HaCaT—human epidermal keratinocyte cells; IL—interleuki; thiobarbituric acid reactive substances (TBARS); human red blood cell (HRBC); TNF-α—tumornecrosis factor-α; COX 2—cyclooxygenase-2; iNOS—inducible nitric oxidesynthase; LPS—lipopolysaccharide; HO-1—heme oxygenase-1; IFN—interferon; ERK—extracellular signal-regulated kinase; MAPKs—mitogen-activated protein kinases; TARC—activation-regulated chemokine; MDC—macrophage-derived chemokine; AMPK—AMP-activated protein kinase; NF-κB—nuclear factor-κB; mTOR—mechanistic target of rapamycin; ROS—reactive oxygen species; HDFa—human dermal fibroblasts adult; NHEK—normal human epidermal keratinocyte; TPC—total phenolic content.

## 6. Encapsulation of Macroalgal Bioactive Compounds: Stabilization Aspects

As discussed in the previous chapter, macroalgae-derived bioactives, including polyphenols, carotenoids, and polysaccharides, possess significant antioxidative, anti-inflammatory properties, which highlight their potential for various applications. However, their application in food and nutraceutical systems is limited by chemical instability, poor dispersibility, and low bioavailability. Most of the BACs are highly sensitive to oxygen, light, temperature, and pH variations, leading to rapid degradation and loss of bioactivity [192]. In addition, many compounds, particularly carotenoids, exhibit poor intestinal absorption due to limited solubility, enzymatic degradation, or low epithelial permeability [12]. Encapsulation thus addresses these challenges by physically isolating the active compound from the external environment, improving dispersibility in food matrices, and enabling controlled release under gastrointestinal conditions [193]. A central insight emerging from the summary of published macroalgal bioactives’ encapsulation studies is shown in Table 2, where encapsulation efficiency and functional performance are strongly dictated by the molecular characteristics of macroalgal bioactives.

The studies on algal polyphenols encapsulation show that encapsulation improves the stability and functional performance of polyphenols, particularly phlorotannins as the most extensively studied ones. In their unencapsulated form, phlorotannins are prone to degradation during processing and digestion, which limits their bioactivity, whereas encapsulation preserves their functionality to a much greater extent [195]. Different delivery systems offer specific advantages depending on the intended application. For example, chitosan-based nanoparticles produced by ionic gelation provide strong protection under storage and gastrointestinal conditions, making them suitable for functional food applications. On the other hand, lipid-based systems such as nanoliposomes improve AOA and dispersion, likely due to better interaction with aqueous environments and increased surface area. An important aspect emerging from these studies is the role of carrier design in controlling release behavior. Secondary coatings, such as alginate–chitosan layers, can slow the release of phlorotannins from liposomal systems, demonstrating that multilayer encapsulation is a useful strategy for tailoring delivery profiles [194]. This emphasizes the importance of carrier architecture when designing targeted delivery systems. In addition, research on *Durvillaea incurvata* shows that encapsulation can be successfully applied in real food systems, enabling the development of stable, bioactive-rich ingredients [196]. This supports the idea that encapsulation is not only a laboratory concept but also a practical tool for food formulation. Despite these advances, the number of primary studies remains limited. Compared to other marine bioactives, such as carotenoids, encapsulation research on macroalgal polyphenols is still relatively underdeveloped and often focuses on model systems or crude extracts. This highlights the need for more systematic research using well-characterized compounds and standardized evaluation methods.

Similarly to polyphenols, a trend is observed in studies on macroalgae-derived pigments, where encapsulation consistently improves stability and functional performance. However, the choice of encapsulation strategy strongly depends on pigment chemistry and the intended application. Carotenoids, which are lipophilic, show good compatibility with lipid-based systems such as nanoemulsions. For instance, flaxseed oil-based nanoemulsions enhance pigment stability and facilitate their incorporation into food matrices [198]. In contrast, chlorophylls are more effectively stabilized using biopolymer-based systems. Fish gelatine and gum Arabic provide high solubility and satisfactory encapsulation efficiency, demonstrating the potential of protein–polysaccharide matrices for chlorophyll encapsulation [199]. Alternative approaches such as the particles from gas saturated solutions (PGSS) technique, applied to *Fucus virsoides* extracts, also show promise. This supercritical CO_2_-based method achieves high encapsulation efficiency while avoiding the use of organic solvents, making it attractive for clean-label and industrial applications [200]. Phycobiliproteins, including phycoerythrin, behave differently due to their water-soluble, protein-based nature. Electrospray encapsulation using alginate systems has been shown to improve both stability and biological activity, including enhanced anticancer potential over time [201]. This suggests that encapsulation can influence not only physicochemical properties but also biological functionality.

Overall, these findings indicate that no single encapsulation strategy is universally optimal. Instead, the choice of carrier and method must be tailored to the physicochemical properties of the compound, particularly its polarity and sensitivity to environmental conditions, as well as the intended application. Polyphenols such as phlorotannins, which are hydrophilic and highly reactive, are best stabilized using polysaccharide- and protein-based systems that enable protection and controlled release during digestion. In contrast, lipophilic pigments such as carotenoids and chlorophylls require lipid-based systems to improve dispersibility and prevent oxidative degradation. The importance of carrier selection is further illustrated by fucoxanthin nanoemulsions, where protein-based emulsifiers like sodium caseinate provide higher encapsulation efficiency than polysaccharides such as fucoidan, while still benefiting from the functional contributions of polysaccharides in modifying viscosity and release behavior [202]. Across both polyphenol and pigment systems, polysaccharides, including alginate, chitosan and fucoidan, play a central and versatile role, acting not only as structural materials but also as active components that influence encapsulation efficiency, stability, and release mechanisms.

In summary, encapsulation represents a powerful and versatile approach for enhancing the stability, bioavailability, and functionality of macroalgal bioactives. However, further research is needed to develop standardized methodologies and to better understand how different encapsulation systems perform across a wider range of compounds and applications as well as to investigate in vivo bioavailability to fully realize the potential of these compounds in functional foods and nutraceuticals.

## 7. Functional Applications in Food and Nutraceutical Industry

**Figure 2:**
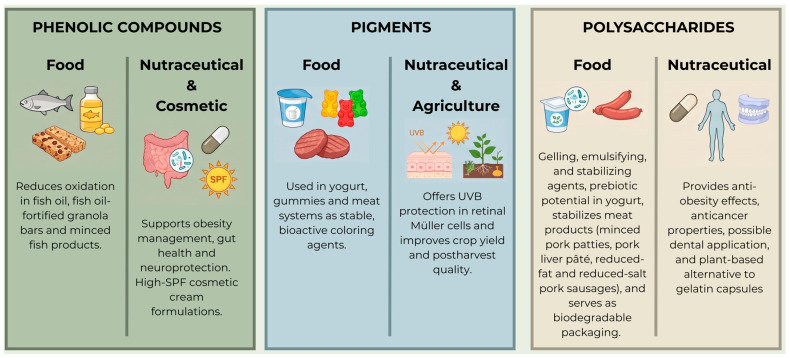
Functional applications of macroalgal bioactive compounds.

The increasing evidence supporting the health-promoting properties of macroalgal BACs, along with advances in extraction and encapsulation technologies, has encouraged their incorporation into a wide range of food and nutraceutical applications (Figure 2).

Phenolic compounds, particularly phlorotannins from brown macroalgae and phenolic-rich extracts from red macroalgae, have been extensively studied for their applications in food systems and emerging nutraceutical formulations. Studies demonstrate their primary role as natural antioxidants, their ability to improve oxidative stability in lipid-rich foods, and their growing relevance in functional food design and oral delivery systems. One of the most established applications of macroalgal phenolic compounds is their ability to inhibit lipid oxidation in complex food matrices. Macroalgal-derived phenolic extracts have been shown to effectively reduce oxidation processes in fish-based systems. For example, New Zealand macroalgal extracts showed strong antioxidant properties, significantly reducing oxidation rates in fish oil and confirming their effectiveness as natural antioxidants in lipid-rich food systems [203]. Similar antioxidant effects were observed in the other study during chilled storage of minced Atlantic mackerel, where macroalgal extracts significantly reduced lipid and protein oxidation, thereby improving product shelf life and stability [204]. This confirms the potential of macroalgal-derived antioxidants to serve as natural preservatives for maintaining the food quality. Another study also reported comparable protective effects of phlorotannins. Wang et al. [205] reported that brown algal phlorotannins, particularly in combination with ascorbic acid, improved the physicochemical stability of minced fish (*Pagrosomus major*) during repeated freeze–thaw cycles by reducing oxidative damage and preserving product quality through the mitigation of lipid oxidation, protein degradation, and texture deterioration. Hermund et al. [206] demonstrated the potential of brown macroalgal antioxidants as natural protective agents in complex food systems by improving the oxidative stability of fish oil-fortified granola bars, reducing lipid oxidation, and preserving product quality during storage. Additionally, Zhang et al. [207] investigated cross-processing strategies that combine fish co-products with agricultural and marine side-streams, including macroalgae, to produce protein isolates with enhanced oxidative stability. They found that co-processing herring and salmon co-products with macroalgae and other side-stream biomass improved the oxidative stability of protein isolates by limiting lipid oxidation.

Beyond technological functionality, macroalgal phenolic compounds demonstrate significant biological activity relevant to nutraceutical development. In particular, phlorotannins modulate fat metabolism and lipid-related pathways, supporting their potential role in obesity management and metabolic health [208]. Kong et al. [209] demonstrated that phlorotannins from the edible brown alga *Ecklonia cava* suppress adipogenesis by reducing lipid droplet formation and downregulating adipogenic markers, highlighting their potential for obesity-related nutraceutical applications. Additionally, phlorotannin-rich extracts from *E. cava* showed neuroprotective effects by reducing oxidative stress-induced cell damage and improving cell viability, supporting their potential application in neuroprotective dietary supplements [210]. Beyond food and nutraceutical applications, phlorotannins have also shown considerable potential in cosmetic and pharmaceutical formulations. A phlorotannin-rich fraction from the brown alga Polycladia myrica exhibited strong antioxidant and antibacterial activities, protected HaCaT keratinocytes against UVB-induced cytotoxicity, and when incorporated into a cream formulation at 5%, exhibited a high sun protection factor (SPF 31.79 ± 4.73) [211].

Recent studies have shown that in vitro digestion and colonic fermentation increase the bioaccessibility and microbial transformation of phenolic compounds from Australian macroalgae while maintaining or enhancing their AOA. These compounds also modulate gut microbiota and promote short-chain fatty acid production, highlighting their potential for gut health and microbiota-targeted nutraceuticals. However, the functionality of macroalgal phenolic compounds is strongly influenced by their interactions with other food components. Phenolic–polysaccharide interactions in brown macroalgae affect the stability, bioaccessibility, and AOA of these compounds, as phenolics can bind to polysaccharide matrices, potentially modulating their release and bioactivity [212].

Besides phenolic compounds, macroalgal pigments, including chlorophylls, carotenoids (such as fucoxanthin), and phycobiliproteins, have gained increasing attention as natural alternatives to synthetic colorants and as functional ingredients in food and nutraceutical formulations. Recent studies demonstrate their dual role as coloring agents and BACs, with applications in dairy products, confectionery, meat systems, and functional foods. Fucoxanthin has been extensively studied for incorporation into functional food systems. For example, encapsulated fucoxanthin improved structural stability and preserved the physicochemical, functional, and bioactive properties of fermented yogurt during refrigerated storage [213]. Similarly, Liu et al. [214] developed a fucoxanthin-enriched macroalga gummy that effectively preserved fucoxanthin stability while maintaining desirable physicochemical properties. In addition, in vitro experiments demonstrated a protective effect against UVB-induced damage in retinal Müller cells, indicating potential bioactivity beyond its nutritional role.

Another study used nanoliposomal delivery systems to enhance the stability and functional performance of fucoxanthin in yogurt. Specifically, Robles-García et al. [215] developed a fucoxanthin-enriched yogurt using nanoliposomal carriers, which improved fucoxanthin stability during processing and storage while preserving the yogurt’s physicochemical properties. The fortified yogurt also showed antioxidant and erythroprotective effects. Overall, these studies collectively demonstrate that fucoxanthin is not only a pigment but also a functional nutraceutical ingredient suitable for incorporation into diverse food matrices when properly stabilized.

On the other hand, chlorophyll-rich macroalgal extracts have primarily been studied in agricultural applications to enhance crop performance and postharvest quality. For example, Mousavi et al. [216] demonstrated that macroalga-based foliar treatments improved the growth, yield, and quality of ‘Golden Delicious’ apple (*Malus domestica*), highlighting the broader functional potential of chlorophyll-containing macroalgal extracts.

Phycobiliproteins, particularly phycoerythrin and related pigments from red macroalgae, have been widely studied as natural food colorants and functional ingredients. Stability and kinetic studies of phycobiliproteins extracted from *Gracilaria gracilis* confirmed their potential for food applications while highlighting the need for stabilization strategies [217]. Chen et al. [218] investigated the potential use of food-grade phycobiliproteins derived from the red macroalga *Porphyra haitanensis* as natural colorants in food systems. The extracted phycobiliproteins showed desirable color characteristics and were effectively encapsulated in liposomes, which improved their stability in a meat-based matrix. Moreira et al. [219] further supported the potential of phycobiliproteins as natural food colorants. Although the phycobiliproteins in their study were derived from the cyanobacterium *Nostoc* PCC9205 rather than macroalgae, the phycobiliprotein-rich extract showed good initial color stability in acidic solutions and yogurt, highlighting the broader applicability of marine-derived phycobiliproteins in food systems.

Another major group of macroalgal BACs comprises polysaccharides, a diverse class of bioactive and functional ingredients that is increasingly used in food and nutraceutical systems. Numerous studies show their role not only as technological additives (such as gelling, emulsifying, and stabilizing agents), but also as BACs with potential health effects when incorporated into real food matrices or oral formulations. Ulvan, a sulfated polysaccharide from *Ulva lactuca* and related green algae, has shown prebiotic potential in synbiotic yogurt systems, supporting its use as a functional food ingredient. For example, Shalaby and Amin [220] demonstrated that ulvan effectively enhanced probiotic survival and activity in synbiotic yogurt while maintaining acceptable physicochemical and sensory properties. Beyond dairy applications, ulvan has been studied in emulsion-based systems for encapsulating and delivering flavor and fragrance compounds. The study by Morelli et al. [221] showed that ulvan can serve as a natural stabilizing and structuring agent in food and cosmetic emulsions, providing good formulation stability and controlled release of volatile compounds. Additionally, Ramu Ganesan et al. [222] demonstrated that ulvan is a promising biopolymer for biodegradable packaging and active food coatings. Ulvan–semi-refined carrageenan composite films showed improved mechanical strength and favorable moisture and gas barrier properties due to interactions between the polysaccharide matrices. Its antioxidant and immunomodulatory activities have also been confirmed in both in vitro and in vivo studies. Garcia-Marquez et al. [223] reported the antioxidant, immunomodulatory, and biocompatible properties of ulvan from *Ulva rigida*, supporting its potential as a nutraceutical ingredient.

Fucoidan and laminarin have been widely studied in food and nutraceutical applications for their bioactive and functional properties. In particular, fucoidan has attracted attention for its metabolic health benefits, with low molecular weight, highly sulfated forms showing anti-obesity effects through modulation of the gut microbiota, supporting its relevance as a nutraceutical ingredient targeting metabolic regulation [224]. This underscores fucoidan’s role not only as a functional food component but also as a BAC with systemic physiological effects beyond technological functionality. In food systems, especially meat products, brown macroalgal extracts containing laminarin and fucoidan have been studied for their ability to enhance product stability through antioxidant and antimicrobial activity. Moroney et al. [225] stated that *Laminaria digitata* extracts reduced lipid oxidation and improved the refrigerated storage stability of minced pork patties, demonstrating protective effects in complex meat matrices. Similarly, dietary supplementation with laminarin- and fucoidan-rich extracts improved the oxidative stability and quality of fresh pork, indicating indirect benefits through the modulation of animal physiology and metabolism [226,227]. Agregán et al. [228] reported that macroalgae extracts from *Ascophyllum nodosum*, *Fucus vesiculosus*, and *Bifurcaria bifurcata* extended the shelf life of pork liver pâté by reducing oxidative degradation and microbial spoilage, highlighting their effectiveness in preserving processed meat. More recently, Mohammed et al. [229] demonstrated that whole brown macroalgae, including *Himanthalia elongata* and *Alaria esculenta*, improved the technological functionality and storage stability of reduced-fat and reduced-salt pork sausages, highlighting the potential of both purified polysaccharides and whole macroalgal biomass in developing healthier and more stable meat products.

Carrageenans from red macroalgae have been studied as hydrocolloids with strong gelling and film-forming properties. Eco-friendly extracted carrageenans formed stable gel networks with appropriate mechanical properties, supporting their use as functional biopolymers in structured food systems. [230] Beyond technological functionality, macroalgal polysaccharides also show significant bioactivity relevant to nutraceutical applications. Monla et al. [231] demonstrated that fucoidan and alginate from brown macroalga *Colpomenia sinuosa* exhibit significant pro-apoptotic activity in colon cancer cells, with enhanced effects when combined with vitamin C. Overall, the findings suggest that these polysaccharides may have promising potential as natural anticancer agents. The versatility of macroalgal polysaccharides was further demonstrated by Hamrun et al. [232], who showed that *Sargassum polycystum* is a promising source of irreversible hydrocolloid impression materials with suitable gel-forming ability, stability, and elasticity for dental applications. Agar polysaccharides have attracted considerable interest as sustainable, plant-based alternatives to gelatin in soft capsule shell formulations [233]. Theological studies of agar hydrogels have demonstrated the feasibility of producing soft capsules from these polysaccharides, which may serve as suitable halal alternatives to conventional gelatin capsules in food and pharmaceutical applications [234,235].

Overall, macroalgae are recognized as a versatile and sustainable source of BACs with significant potential for integration into food and nutraceutical systems. Their role has evolved from traditional functional additives to multifunctional ingredients that provide both technological performance and health-promoting properties. The increasing use of phenolics, pigments, and polysaccharides in real food matrices highlights their potential to support the development of clean-label, health-oriented, and sustainable products. However, several scientific, technological, and regulatory challenges must still be addressed before their full industrial potential can be realized.

## 8. Challenges, Regulatory Aspects, and Future Perspectives

Although macroalgal BACs have considerable potential for functional food and nutraceutical applications, broader industrial use remains limited by variability in raw materials, challenges with compound stability and bioavailability, the complexity of extraction and scale-up processes, and differing regulatory requirements across markets. Addressing these challenges is essential for successful commercialization and wider adoption of macroalgae-derived ingredients.

### 8.1. Technological and Production Challenges

As previously outlined, a major challenge in developing products from marine BACs is the high variability in the chemical composition of raw materials. The content and profile of BACs in macroalgae depend significantly on season, geographical origin, sea temperature, salinity, light conditions, and the nutritional status of the environment [29,236,237]. This variability makes it difficult to standardize extracts and ensure consistent product quality, which is crucial for industrial applications and regulatory approval. A potential approach to overcoming this challenge is the implementation of controlled cultivation systems and standardized harvesting conditions, combined with advanced analytical monitoring and blending strategies to ensure more consistent chemical composition of the raw materials and final extracts. Scaling up extraction technologies represents another significant challenge. Although advanced extraction methods are highly efficient at laboratory scale, their industrial application is often limited by high investment costs, the need for specialized equipment, and complex process control. In addition, process optimization must consider not only yield and selectivity but also energy efficiency and environmental impact. To address these challenges, developing cost-effective scale-up strategies, combined with energy-efficient and environmentally sustainable technologies, are necessary to enable the industrial implementation of advanced extraction methods. Maintaining the stability of BACs during processing, storage, and distribution also remains a critical concern [238]. Many marine BACs, such as polyphenols and carotenoids, are highly susceptible to oxidation, light, heat, and changes in pH. Without appropriate technological approaches, such as encapsulation or mild processing methods, loss of bioactivity can significantly reduce the functional value of the final product [239,240].

### 8.2. Regulatory and Security Aspects

Beyond technological and production challenges, regulatory compliance and product safety requirements are essential for the successful commercialization of marine BACs. In the European Union (EU), algal products intended for food and food supplement applications are subject to the Novel Food Regulation (EU) 2015/2283 if they lack a documented history of significant consumption within the EU before 15 May 1997 [241]. Therefore, algal species, extracts, or isolated BACs-considered novel foods must undergo a scientific safety assessment as part of the authorization procedure before being placed on the market [242]. As part of this process, the European Food Safety Authority (EFSA) conducts an independent scientific risk assessment to support the authorization procedure carried out by the European Commission. This ensures that marine-derived BACs meet strict safety standards before commercial use. In this context, the safety assessment of algal products covers not only their suitability for human consumption but also compliance with legal requirements, accurate labeling, product traceability, and a stable, controlled supply chain [243]. While the Novel Food Regulation serves as the central regulatory mechanism for novel algal products, marine BACs are also subject to a broader framework of EU food legislation designed to ensure consumer protection, product quality, and market transparency. These frameworks address several critical areas, including authorization procedures for novel ingredients, substantiation of nutrition and health claims, labeling requirements, and general food safety principles. Depending on their source, composition, and intended application, marine BACs (such as carotenoids, polysaccharides, and algal oils) may also fall within the scope of food additive legislation or the Food Supplements Directive (2002/46/EC) [244]. A summary of the key EU regulations relevant to the development, commercialization, and safe use of marine BACs is provided in Table 3. The Novel Food Regulation also provides a mechanism to recognize certain algae as traditional foods when there is documented evidence of safe consumption in any part of the world for at least 25 years, such as in various Asian cultures. This procedure may facilitate market entry by reducing regulatory requirements and simplifying the authorization process [245]. Despite harmonized EU legislation, the implementation and interpretation of algal regulations may vary among member states. National rules or guidelines may apply to specific species, particularly those with a history of traditional consumption only in certain regions [245].

These examples show how the EU novel food system explicitly addresses marine BACs, requiring each new source or extract to be individually evaluated and authorized before commercialization. Even compounds with established nutritional value, such as omega-3-rich oils from algae or krill, must comply with rigorous safety assessments and labeling requirements before entering the European market.

### 8.3. Future Perspectives and Research Directions

While regulatory compliance and safety assessment remain essential prerequisites for market approval of marine BACs, ongoing technological and scientific advances are creating new opportunities to overcome existing limitations and expand their industrial applications. Marine BACs show considerable potential for use in the health, cosmetic, and functional food sectors while advances in sustainable algae aquaculture and modern biotechnological cultivation strategies provide a reliable and controlled source of these valuable compounds and help reduce raw material variability. Nevertheless, several key challenges must still be addressed to facilitate the broader industrial application of marine macroalgae. These include further optimization of cultivation systems, enhancement of biomass productivity and metabolite yields, reduction in downstream processing costs, and effective compliance with increasingly complex regulatory frameworks. In addition, the lack of standardized BAC profiles, limited validation of biological efficacy, and challenges associated with large-scale production continue to constrain wider industrial implementation [255]. Advances in encapsulation technologies and intelligent delivery systems are expected to improve the stability, bioavailability, and targeted delivery of marine algal BACs. Furthermore, combining marine BACs with other functional ingredients, such as dietary fiber, vitamins, or probiotics, offers promising opportunities for the development of innovative products with synergistic health benefits [13]. Future research should focus on elucidating the mechanisms of action of marine BACs, standardizing extraction procedures and product composition, and conducting well-designed preclinical and clinical studies to confirm their safety and efficacy. An interdisciplinary approach integrating food science and technology, biotechnology, nutrition, pharmacology, and regulatory sciences will be essential for translating scientific knowledge into safe, effective, and commercially viable products. Overall, despite the remaining scientific, technological, and regulatory challenges, continued advances in biotechnology, cultivation systems, extraction technologies, and delivery strategies are expected to strengthen the commercial viability and industrial scalability of marine BACs. Alongside the growing consumer demand for natural, sustainable, and health-promoting products, these developments position marine-derived BACs as promising ingredients for future applications in the food, nutraceutical, and cosmetic sectors.

## 9. Conclusions

Macroalgal BACs are a diverse and valuable group of natural substances with significant potential for applications in the food and nutraceutical industries. As highlighted in this review, their chemical diversity, including polyphenols, pigments, and polysaccharides, supports a wide range of biological activities, particularly antioxidant and anti-inflammatory effects, making them promising functional ingredients for health promotion and disease prevention. The effective use of these bioactives depends not only on identifying suitable biomass sources but also on developing efficient cultivation, extraction, and processing strategies that preserve their bioactivity and ensure consistent product quality. Because the composition of macroalgal BACs is strongly influenced by environmental and seasonal factors, establishing standardized production and characterization protocols are essential for reliable industrial application. Furthermore, continued advances in encapsulation technologies are essential for improving the stability, bioavailability, and controlled delivery of macroalgal BACs, thereby maximizing their functionality in food and nutraceutical applications. Although challenges related to large-scale production, standardization, regulatory compliance, and market acceptance persist, ongoing progress in marine biotechnology, sustainable cultivation systems, extraction technologies, and product formulation continues to expand opportunities for commercial utilization. Overall, macroalgae represent a sustainable and highly promising source of health-promoting BACs, and continued interdisciplinary research and collaboration among academia, industry, and regulatory authorities will be crucial for translating their biological potential into safe, effective, standardized, and commercially viable products, ultimately supporting the development of innovative functional foods and nutraceuticals.

## Figures and Tables

**Table 2:** Overview of encapsulation approaches for macroalgae-derived polyphenols and pigments.

Class	Compound	Source	Method	Carrier	Key Outcome	Reference
Polyphenols	Phlorotannins	*Sargassum tenerrimum*	Nanoliposomal encapsulation	Liposomes (±alginate–chitosan coating)	Encapsulation significantly increased AOA and stability; coating slowed release	[194]
	Phlorotannins	*Sargassum ilicifolium*	Ionic gelation nanoparticles	Chitosan–tripolyphosphate	Improved storage stability, processing stability, and retained bioactivity after digestion	[195]
	Phenolic extract	*Durvillaea* *incurvata*	Spray drying	Maltodextrin	Improved stability and enabled development of functional ingredient with anti-inflammatory activity	[196]
	Phenolic extract	*Sargassum ilicifolium*	Freeze-drying microencapsulation	WPI + maltodextrin/chitosan	Up to ~99% encapsulation efficiency; improved stability and solubility	[197]
Pigments	Carotenoids	*Gracilaria dura*, *Sargassum* *acinarium*, *Ulva rigida*	Nanoemulsions	flaxseed oil	Enhanced stability and suitability for food systems	[198]
	Chlorophyll	*Caulerpa* *racemosa*	Freeze drying	fish gelatine and Arabic gum	High solubility of microcapsules with satisfying encapsulation efficiency	[199]
	Carotenoid and chlorophyll rich supercritical CO_2_ extract	*Fucus* *Virsoides* J. Agardh	Particles from gas-saturated solution (PGSS) process	Polyethylene glycol	High encapsulation efficiency	[200]
	Phycoerythrin	*Porphyridium purpureum*	Electrospray encapsulation	Alginate, Alginate-Maltodextrin, Alginate-Pectin, Alginate-Arabic gum	Enhanced in vitro anticancer activity over time	[201]
	Fucoxanthin	*Sargassum angustifolium*	Nanoemulsions	black seed oil + fucoidan, gum Arabic, and sodium caseinate	The protein-based emulsifier showed better function; fucoidan and gum Arabic exhibited a relatively high encapsulation efficacy and fucoxanthin release	[202]

Abbreviation: AOA—antioxidant activity.

**Table 3:** EU regulatory frameworks relevant to marine-derived bioactive compounds.

Regulation/Directive	Scope and Relevance	Example of Marine Bioactive Compounds	References
Regulation (EU) 2015/2283—Novel Food Regulation	The central EU framework governing foods or ingredients that had no significant history of consumption before 15 May 1997. It requires a safety assessment by the European Food Safety Authority (EFSA) and authorization before products can be placed on the market.	*Odontella aurita* microalgae (authorized entry on the Union list); algal meal from *Haematococcus pluvialis* containing astaxanthin (EFSA safety assessment).	[246,247]
Commission Implementing Regulation (EU) 2017/2470—Union List of Novel Foods	Establishes the official list of authorized novel foods within the EU and specifies conditions of use, labeling requirements, and specifications.	*Odontella aurita* microalgae included with defined conditions of use.	[246]
Regulation (EC) No. 1924/2006—Nutrition and Health Claims Regulation	Regulates the use of nutrition and health claims for foods and food supplements, requiring scientific substantiation and approval of claims.	Claims related to omega-3 fatty acids from algae or marine oils require scientific validation; health claims for certain algal fibers have not been authorized due to insufficient characterization.	[248,249]
Directive 2002/46/EC—Food Supplements Directive	Harmonizes the definition and regulatory requirements for food supplements within the EU.	Marine oils rich in EPA and DHA used in dietary supplements; microalgal omega-3 fractions incorporated into supplements.	[244,250,251]
Regulation (EU) No. 1169/2011—Food Information to Consumers (FIC)	Establishes rules for clear and accurate food labeling, ensuring that consumers receive understandable information across EU Member States.	Labeling of algal oils, omega-3 sources, and marine-derived ingredients must comply with naming and nutritional declaration requirements.	[252]
Regulation (EC) No. 178/2002—General Food Law	The foundational EU regulation establishing general principles of food safety, traceability, and responsibilities of food business operators.	Ensures that marine-derived bioactive ingredients placed on the market do not pose a risk to consumer health.	[253]
Regulation (EC) No. 1333/2008—Food Additives Regulation	Governs the use of technological food additives in food products within the EU.	Applicable when marine bioactive compounds are used as additives, such as antioxidants or stabilizers (e.g., algal carotenoids in oils).	[254]

## Data Availability

No new data were created or analyzed in this study. Data sharing is not applicable to this article.
